# Development of brain metastable dynamics during the equivalent of the third gestational trimester

**DOI:** 10.1016/j.dcn.2025.101556

**Published:** 2025-04-07

**Authors:** Juliette L.Y. Champaud, Samanta Asite, Lorenzo Fabrizi

**Affiliations:** a Department of Neuroscience, Psychology and Pharmacology, University College London, UK; b Centre for the Developing Brain, King’s College London, UK

**Keywords:** Metastability, Neonatal brain development, Brain functional dynamics, Cognition, Sensory processing, Preterm

## Abstract

Metastability, a concept from dynamical systems theory, provides a framework for understanding how the brain shifts between various functional states and underpins essential cognitive, behavioural, and social function. While studied in adults, metastability in early brain development has only received recent attention. As the brain undergoes dramatic functional and structural changes over the third gestational trimester, here we review how these are reflected in changes in brain metastable dynamics in preterm, preterm at term-equivalent and full-term neonates.

We synthesize findings from EEG, fMRI, fUS, and computational models, focusing on the spatial distribution and temporal dynamics of metastable states, which include functional integration and segregation, signal predictability and complexity. Despite fragmented evidence, studies suggest that neonatal metastability develops over the equivalent of the third gestational trimester, with increasing ability for integration-segregation, broader range of metastable states, faster metastable state transitions and greater signal complexity. Preterms at term-equivalent age exhibit immature metastability features compared to full-terms. We explain and interpret these changes in terms of maturation of the brain in a free energy landscape and establishment of cognitive functions.

## 1 Introduction

The brain is never truly at rest: even in the absence of external stimuli or tasks, it remains active, continuously generating patterns of electrical and hemodynamic activity (Raichle, 2015, Shoham et al., 2006). This ongoing activity is not random but instead exhibits meaningful, structured patterns, which reflect the brain's intrinsic organization (Raichle, 2015). To uncover these patterns, we must move beyond static measurements of brain activity, which offer a limited, snapshot-like perspective. Traditional approaches, such as functional connectivity (FC), are important for understanding the brain's architecture and baseline function (Park and Friston, 2013), however, they do not capture the inherently time-varying nature of neural processes (Grosu et al., 2023) and cortico-cortical interactions (Park and Friston, 2013, Kelso, 2012). The brain is indeed a dynamic system that can be described as *metastable* (Tognoli and Kelso, 2014). Metastability is a concept originating from dynamical systems theory and refers to a system poised between stability and instability (Deco et al., 2017). It is characterized by transient occupations of equilibrium points, followed by transitions between them (Cavanna et al., 2018). In the brain, this manifests as a balance between periods of coordinated activity and flexible shifts to new configurations (Jang et al., 2024). These transient configurations of activity, i.e. equilibrium points, whether at large or small spatial or temporal scale, are denoted as *metastable states*. Brain metastability in adults has been already reviewed many times so we redirect the reader to other excellent reviews on the subject e.g. (Tognoli and Kelso, 2014, Brinkman et al., 2022, Kelso, 2012, Hancock et al., 2025).

Metastability represents the ability of the brain to balance brain-wide cooperation (integration), while retaining functional specificity (segregation) over time (Tognoli and Kelso, 2009, Jang et al., 2024). This balance enables flexible information routing for real-time adaptation to changing internal and external demands, which is crucial for perception, memory, decision-making, and other cognitive functions (Alderson, Thomas et al., 2020, Kelso, 2012; L. Mazzucato et al., 2019; Rabinovich et al., 2008). This adaptability ensures that the brain can allocate resources efficiently, shift between modes of processing, and maintain a coherent, task-relevant network configuration (Wang et al., 2021; Jang et al., 2024). Markers of metastability have thus been employed to explore various aspects of brain function, such as cognitive performance (higher performance associated with more flexible integration-segregation) (Alderson et al., 2020), aging (characterised by higher signal variability and more fragmented metastable transitions) (Naik et al., 2017, Davison et al., 2016), meditation and sleep (during which metastability is altered resulting in reduced flexibility) (Wiemers et al., 2023, Escrichs et al., 2019), and to characterize psychiatric conditions or neurological disorders (schizophrenia and Alzheimer for example are associated with an imbalance of functional segregation and integration) (Hancock et al., 2023, Córdova-Palomera et al., 2017, Alderson et al., 2018), advancing our understanding of neural circuits in health and disease.

**Fig. 1:**
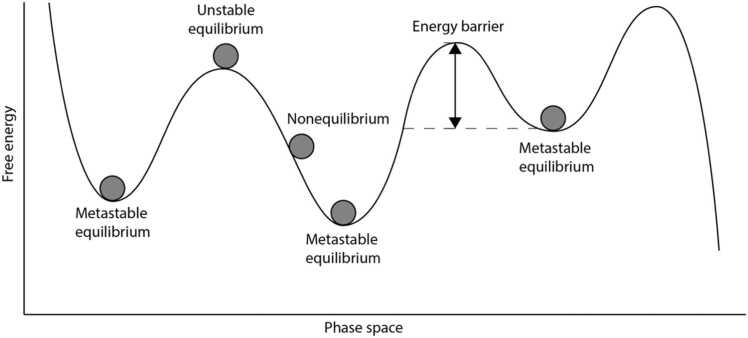
Illustration of a metastable system. Brain activity is represented by a “sphere” moving across a free energy landscape with wells and barriers. The wells represent local energy minima and therefore metastable equilibrium points. The system can leave a metastable state only when enough energy is available to overcome the energy barrier. The shallower the wells the more unstable the metastable states.

The metastable brain is often conceptualised as a “sphere” moving in a free energy landscape (Fig. 1). Free energy refers to the energy available to the brain (Friston, Kilner, and Harrison, 2006), which ultimately relates to the metabolic cost of establishing and maintaining functional activity (e.g. synaptic activity and ion transportation) (Harris et al., 2012, Friston, 2010). This landscape is characterised by local wells of low energy, which represent equilibrium points (i.e. the metastable states). Provided with sufficient energy, the brain can “move” between these equilibrium points by overcoming energy barriers between them (Cavanna et al., 2018, beim Graben et al., 2019). Energy barriers can be understood as a combination of functional and structural constraints that impede transitions between metastable states. These include the physical architecture of the brain (e.g., white matter connectivity), the intrinsic stability of functional networks and the balance of excitation and inhibition activity (Uchitel et al., 2021, Wu et al., 2024, Desrosiers et al., 2024; Y. Ben-Ari, 2014). The connectivity architecture defines transitions more or less energetically costly through more or less well-connected pathways (Sun et al., 2023, Iraji et al., 2019), while the homeostatic excitation-inhibition balance allows the brain to sustain transient activations (metastable states) without collapsing into persistent synchronization or complete desynchronization (Fontanier et al., 2022; Santos and Verschure, 2024). Overcoming these barriers requires sufficient perturbation (e.g., task demands, environmental stimuli, or intrinsic fluctuations) to destabilize the current metastable state and enable a shift to a new functional configuration.

These metastability-defining infrastructures mature dramatically over the third gestational trimester and are affected by preterm birth (Doria et al., 2010, Batalle et al., 2017, Eyre et al., 2021, Cao et al., 2017, Ball et al., 2013; Ben-Ari, 2001; Allene and Cossart, 2010; Murata and Colonnese, 2020). More specifically, the third gestational trimester sees rapid growth and refinement of the cortical structure including the expansion of subplate zones (Ivica Kostović and Jovanov-Milošević, 2006), dendritic differentiation, synaptogenesis and myelination (I. Kostović, Sedmak, and Judaš 2019) that promote the establishment of structural connections and networks (Batalle et al., 2017; Van Den Heuvel et al., 2015). Functional organisation also matures with the emergence of resting state networks (RSNs) such as the default mode (DMN), sensorimotor and visual networks (Doria et al., 2010). Additionally, there is a shift in the excitation-inhibition balance, moving from excitation dominance toward a more balanced homeostasis (Yehezkel Ben-Ari, 2001; Owens et al., 1999), potentially due to a transition in GABA function from depolarizing to hyperpolarizing (Murata and Colonnese, 2020), along with an increase in extracellular GABA concentration (Basu et al., 2020).

Preterm neonates at term-equivalent show reduced structural connectivity of white matter in cortico-cortical and thalamo-cortical tracts (Batalle et al., 2017, Ball et al., 2013) as well as delayed emergence and reduced functional connectivity in RSNs (Doria et al., 2010) and inter-hemispheric and long-range connections (Eyre et al., 2021), leading to an overall impaired functional network segregation (Cao et al., 2017). The excitation-inhibition balance may also be affected by premature birth. While GABAergic interneuron activity has not been directly studied in preterms at term-equivalent, some evidence shows that preterm birth is associated with fewer active GABAergic interneurons (Stolp et al., 2019, Lacaille et al., 2019), and reduced expression of GABA receptor subunits (Shaw et al., 2015). Findings on extracellular GABA levels in preterm at term-equivalent compared to full-term controls are mixed (Basu et al., 2021), with some studies reporting reduced levels in preterms at term-equivalent (Kwon et al., 2014), while others observe no significant differences (Kreis et al., 2002).

Understanding how brain metastability is affected by the structural and functional changes occurring over the equivalent of the third gestational trimester will help in evaluating the early establishment of cognitive functions and their potential impairments. Prematurity affects 10 % of neonates worldwide (Ohuma et al., 2023), insights into metastability can improve our understanding of the neurodevelopmental consequences of preterm birth, potentially informing interventions aimed at mitigating cognitive and behavioural outcomes.

In this review, we first divided the tools used to study metastability into three categories: (A) spatial distribution of metastability, (B) temporal dynamics of metastability and (C) signal complexity. Next, we review how structural and functional brain development is reflected in changes in these three aspects of metastability and how these are affected by different behavioural states and following external perturbation. We provide interpretations of this development in the framework of the metastable free energy landscape and in terms of functional relevance to cognitive development.

## 2 Brain metastability – concepts and metrics

Metastability, encompassing spatially and temporally structured patterns of functional brain activity, can be described using different neuroimaging modalities such as functional magnetic resonance imaging (fMRI), electroencephalography (EEG) and functional ultrasound (fUS). Metastability features can be extracted calculating various metrics from the time-series related to these acquisition modalities.

### 2.1 Spatial distribution of metastability

One of the core features of metastability is the characterisation of metastable states in terms of spatial distribution of signal magnitude (amplitude or power) or connectivity at different temporal scales. We define each type of metastable state and describe how they are extracted from neuroimaging time-series in Table 1.

**Table 1:** Classification of metastable states according to modalities, measures, duration and analysis methods.

**Metastable State Name**	**Imaging Modality**	**Measure**	**Duration**	**Analysis Method**	**References**
**Microstates**	EEG	Magnitude	100 ms	Spatiotemporal clustering of quasi-stable EEG topographies based on spatial voltage patterns	Hermans et al. (2024)
**HMM States**	EEG	Connectivity/Magnitude	100 ms	Hidden Markov Models (HMMs) applied to EEG time-varying connectivity matrices or power distributions	Khazaei et al. (2023)
**dFC States**	fUS	Connectivity	1 second	Temporal clustering of fUS dynamic functional connectivity (dFC) matrices	Baranger et al. (2021)
**dFC States**	fMRI	Connectivity	10 seconds	Temporal clustering of fMRI dynamic functional connectivity (dFC) matrices	Ma, Wu, and Shi, (2020); França et al. (2024)

The varying spatial distribution of connectivity-based metastable states reflects changes in functional segregation and integration across the brain. A popular tool for studying this feature of metastability is the Kuramoto model which describes the capacity of the brain to span different levels of synchrony (Kuramoto and Kuramoto, 1975). The Kuramoto model defines time-series acquired from different brain regions with, for example fMRI or EEG, as interconnected oscillators, each defined by a phase and an intrinsic natural frequency. When these time-series are out of phase, the system is segregated, when time-series are in phase, the system is integrated. The global level of phase synchronicity at any point in time is measured with the Kuramoto order parameter (KOP) (Box 1). The mean KOP characterises the overall phase synchronization of the system and KOP standard deviation (std) provides a measure of how synchronicity between brain regions fluctuates over time and is used as a proxy for metastability (Y. Kuramoto, 1984; Breakspear, Heitmann, and Daffertshofer, 2010). Higher KOP std means that the consistency of the phase of the time-series from different brain regions has a wider range, fluctuating between periods of independent with periods of cohesive oscillations. It translates into a larger range of metastable states due to more possible configurations.

Box 1 **Calculating KOP: Example and Equation.**

Taking *N* fMRI BOLD time-series from different brain regions, KOP assesses the time-varying phase synchronicity between each time-series *j* with respective phase *θ* at given time *t* (Eq. 1) (Y. Kuramoto, 1984).

$\mathrm{KOP}\left(t\right)=\;\left|\frac{1}{N}\sum_{j=1}^{N}e^{i\theta_{j}\left(t\right)}\right|$(1)

### 2.2 Temporal dynamics of metastability

Classical measures of temporal behaviour of metastable states include duration, occurrence frequency, coverage and syntax. Duration quantifies how long a metastable state persists before transitioning (Khanna et al., 2015). Longer durations may indicate greater stability of functional brain networks, which can support sustained cognitive processes such as attention or working memory. In contrast, shorter durations may reflect increased flexibility, allowing the brain to rapidly adapt to environmental demands (Tarailis et al., 2023). Occurrence frequency reflects how often a particular metastable state appears, providing insights into the relative prominence of specific functional networks and their engagement in ongoing tasks (Michel and Koenig, 2018, Khanna et al., 2015). These two measures combined indicate the transition rate of the metastable state sequence, describing the speed and flexibility with which the brain functions (Zanesco, 2024). Coverage measures the proportion of time each metastable state occupies relative to the total recording, offering an index of the overall dominance or accessibility of certain functional networks (Murray et al., 2008). The temporal syntax of metastable states represents the structured, time-dependent patterns of state transitions and durations, such as whether there is a consistent sequence of state appearances or a most likely transition from one state to another. It is characterized through transition probabilities, which quantify the likelihood of moving from one metastable state to another (Lehmann et al., 2005). Preferential transitions between metastable states may reflect optimized pathways for efficient information processing, while disrupted or random transitions could indicate developmental immaturity or pathology.

### 2.3 Signal complexity

Another index to characterise a metastable system is its entropy, which provides a quantitative measure of the disorder in a system. It is inversely related to predictability: future data points are relatively easy to anticipate in a tidy ordered time-series (low entropy) which renders it highly predictable (Lau et al., 2022). Entropy is also often associated to information content: a time-series defined by many components is more likely to be disordered and therefore requires more information to describe it (Shannon, 1948). Linked to information content is complexity, a distinct but intertwined measure to entropy (H. Wang et al., 2024). Complexity has many different definitions but, in this review, we define it as a measure of the richness of brain activity—how many different patterns of activity exist and how they interact (Bassett and Gazzaniga, 2011). The assumption is that the more parts in a signal (high complexity), the higher the unpredictability and the more information content in its behaviour (high entropy) (Nezafati et al., 2020, Lau et al., 2022).

Levels of entropy are calculated from time-series acquired from specific brain regions with, for example, fMRI or EEG. There are multiple types of quantifiable entropy, however in this review we focus on two types: sample entropy (SE) and multiscale entropy (MSE) (Box 2). Both are used as measures of predictability and complexity.

Box 2 **Calculating Entropy: Example and Equation.**

Taking as an example fMRI BOLD time-series of length *N*, SE (Eq. 2) assesses whether the similarity, within a tolerance *r*, between time-series’ segments of length *m* persists for time-series’ segments of length *m+ 1* (Richman and Moorman, 2000). A conditional probability is calculated as the ratio of the number of pairs of segments of length *m+ 1* that are similar (A) – measured using any distance function e.g. Euclidean – over the number of pairs of segments of length *m* that are similar (B). B will always be larger than A therefore the ratio will always be between 0 (i.e. the similarity between segments of length *m* completely drops for segments of length *m+1* therefore the time-series can be explained by many different components reflecting high information content) and 1 (i.e. opposite effect, information content is low in this case). To limit the exponential effect whereby an increase in number of components making up a time-series results in an exponential increase in possible interactions i.e. information content, the natural logarithm is used. To always restrict SE to a positive number, it is calculated as the negative natural logarithm. MSE extends the concept of SE by performing the above calculations across multiple temporal scales (Costa et al., 2005). This is achieved by coarse-graining the time-series to create successively lower-resolution versions and then calculating SE for each timescale.

$\mathrm{SE}\left(m,r,N\right)=\;-\ln\left(\frac{A}{B}\right)$(2)

Finally, we introduce scale-invariance as an additional feature linked to metastability. A scale-invariant signal reflects long-term correlations and persistent activity, which displays capacity for functional integration and indicates a complex yet ordered signal (Mella et al., 2024, Grosu et al., 2023). Scale-free properties are defined by power-law functions, $y=x^{\alpha }\;$ where x can represent: (i) oscillation frequency and y power (or amplitude); (ii) spatial spread or duration in time and y probability of occurrence and (iii) timescales and y variability of the signal. α always represents the slope of these logarithmic relationships and it is negative in (i) and (ii), but positive in (iii). This means that: (i) low-frequency events have more power than those with fast oscillations (Freeman and Zhai, 2009, Linkenkaer-Hansen et al., 2001) and that (ii) the probability of occurrence of small-scale (in time and space) events is higher than widespread, long-lasting events (Beggs and Plenz, 2003). In (iii) alpha is called Hurst exponent, which reflects an increase in the standard deviation of the residuals (or root mean square deviation) around a piece-wise linear fit to the signal split in segments of different lengths (i.e. timescales) (Peng et al., 1995, Linkenkaer-Hansen et al., 2001). A small Hurst exponent (<0.5) indicates that the standard deviation of the residuals stays relatively similar whether the piece-wise fit is made on short or long segments (the signal is completely anti-persistent - i.e. values are likely to reverse direction between successive samples - and there is no trend at any timescale). An exponent value > 0.5 on the other hand signifies a steeper increase in the standard deviation of the residuals as the length of the segments increases i.e. the way fluctuations increase with scale is consistent, reflecting scale-invariance and long-term correlations (Van De Ville et al., 2010).

In summary, entropy and scale invariance metrics provide a measure of the amount of information contained in the signal from a single brain region or single time-series across multiple temporal scales. This translates to indirectly assessing the range of available metastable states (due to more or less contained information) coexisting at different temporal scales, or the predictability and complexity of a sequence of metastable states.

## 3 Neonatal development of brain metastability

In this section, we organised evidence of brain metastability development in the three previously described categories. We report evidence of developmental changes during the equivalent of the third gestational trimester (i) comparing preterms (27–37 weeks postmenstrual age (PMA)) to full-terms (37 + weeks PMA), (ii) following preterms longitudinally (from 27 to 37 + weeks PMA) and (iii) comparing preterms at term-equivalent to full-terms.

### 3.1 Spatial distribution of neonatal metastability

**Fig. 2:**
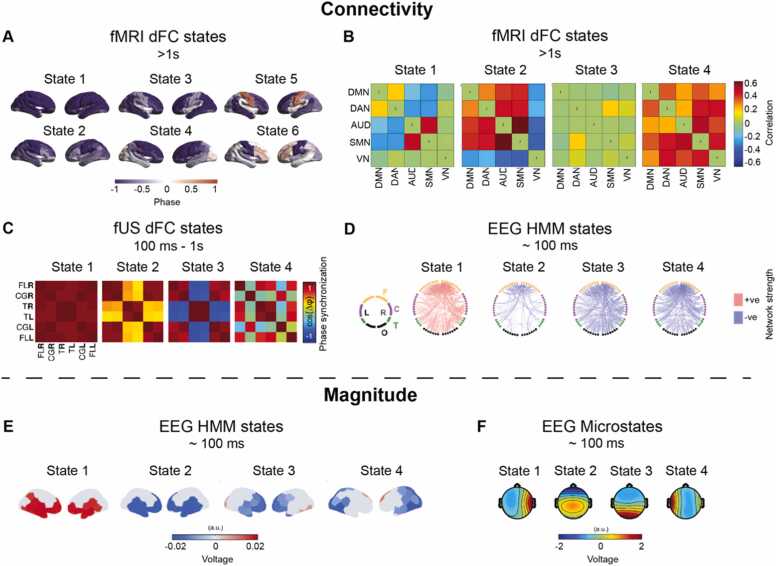
Metastable states characterised by signal connectivity or magnitude at varying spatial and temporal scales defined in the equivalent of the third gestational trimester. A – fMRI dFC states across preterms at term-equivalent and full-terms, displaying spatial patterns of phase synchronization across brain regions (same-coloured regions are more in-phase with each other) adapted from (França et al., 2024). B – fMRI dFC states across preterms at term-equivalent and full-terms, characterised by correlation between RSNs (DAN: dorsal attention network, AUD: auditory network, SMN: sensorimotor network, VN: visual network) adapted from (Ma et al., 2020). C – fUS dFC states across preterms and full-terms characterised by phase synchronization between six brain areas (R: right, L: left, FL: frontal lobe, CG: cingulate gyrus, T: thalamus) adapted from (Baranger et al., 2021). D – EEG HMM states of connectivity between brain regions (F: frontal, C: central, T: temporal, O: occipital) in full-terms, displaying connectivity strength above (red) and below (blue) the mean across all metastable states adapted from (Khazaei et al., 2023). E – EEG HMM states of signal magnitude in each brain region in full-terms adapted from (Khazaei et al. 2023). F – EEG microstates in preterms adapted from (Hermans et al., 2024).

Metastable states are defined in terms of spatial distributions of signal magnitude or connectivity (Fig. 2). The temporal scale, from milliseconds (ms) to seconds, varies depending on the method used to measure them (Table 1). EEG metastable states last 100 s of milliseconds and have been defined in terms of spatial distributions of signal magnitude or connectivity (Pascual-Marqui et al., 1995, Chen et al., 2024). EEG activity magnitude can be clustered into 4 Hidden Markov Model (HMM) states (Khazaei et al., 2023) or 7 microstates (Khazaei et al., 2021) in full-terms, while preterms exhibit 4 microstates (Hermans et al., 2024). Preterms at term-equivalent have been reported to have less (4) or the same number of microstates (7) compared to full-terms (Hermans et al., 2024, Parvaneh et al., 2025). EEG connectivity can be clustered into 4 HMM states in full-terms (Khazaei et al., 2023). FUS dynamic functional connectivity (dFC) states last about one second, also represent clusters of connectivity distributions, and are 4 across preterms and full-terms (Baranger et al., 2021). FMRI dFC states last tens of seconds, represent clusters of connectivity distributions, and are limited to 4–6 across preterms at term-equivalent and full-terms (França et al., 2024, Ma et al., 2020). The balance between segregation and integration is represented by the different connectivity distributions of metastable states. For example, metastable state 1 in Fig. 2A, C and D, where the connectivity between different areas is homogeneous, represent a functionally integrated brain, while other metastable states such as 5 in Fig. 2A, 2 in Figs. 2B and 4 in Fig. 2C, where the connectivity between different areas is more fragmented, represent functionally segregated activity.

A summary index of this balance is KOP (Eq. 1) std. There is no evidence of difference in KOP std between preterms and full-terms. However, preterms at term-equivalent exhibit lower KOP std compared to full-terms, as measured in fMRI BOLD time-series (França et al., 2024). This finding aligns with the notion that preterm birth disrupts the typical trajectory of dynamic network development, leading to reduced variability in functional connectivity states.

In summary, the range of metastable states varies, depending on the metastable state type, during the equivalent of the third gestational trimester. The range of EEG microstates increases indicating brain activity with higher information content at fast temporal scales. Microstates have been given distinct functional roles; therefore this maturational change might relate to a greater range of cognitive abilities (Britz et al., 2010). This developmental trend is not present at slower temporal scales (fUS) and might depend on the different physiological underpinning metastable states defined with different modalities (e.g. electrophysiological vs hemodynamic and magnitude vs connectivity).

It is unclear whether preterms catch up with full-terms by term-equivalent age or have a narrower range of metastable states across timescales/imaging modalities, but they do span a limited spectrum of synchronization levels (lower KOP std, meaning narrower variations between integration and segregation). A broader spectrum of synchronization levels is typically associated to cognitive performance in adults (Padilla et al., 2023, Alderson et al., 2018) and could potentially mean that the cognitive repertoire is impacted by preterm birth.

### 3.2 Temporal dynamics of neonatal metastability

In preterms, the brain spends more time in (i) a fUS dFC state (higher coverage) characterised by strong intra-thalamic and intra-cortical and weak thalamo-cortical connections compared to full-terms and less in a metastable state of global synchronization (Baranger et al., 2021) and (ii) an EEG microstate representing a strong posterior voltage positivity (Hermans et al., 2024). In terms of syntax, the likelihood of transitioning into this EEG microstate decreases in preterms followed longitudinally (Hermans et al., 2024). Dominant coverage of fMRI dFC states characterised by global synchrony and local sensorimotor synchrony increases postnatally in full-terms over the first week after birth (França et al., 2024, Ma et al., 2020) and metastability syntax includes more likely transitions to fMRI dFC states covering areas associated with the DMN (França et al., 2024). Compared to full-terms, preterms at term-equivalent occupy more fMRI dFC states characterised by weak connectivity between RSNs (DMN, dorsal attention, auditory, sensorimotor, visual) to the detriment of whole-brain synchronization (Ma et al., 2020) with a preference for those involving local occipital and frontoparietal (França et al., 2024) but not sensorimotor-auditory synchronization (Ma et al., 2020).

In summary, coverage of globally synchronized and DMN-related metastable states increases during the equivalent of the third gestational trimester. The DMN is a core attractor, acting as a transition hub and providing momentary stability or “reset” between metastable states to facilitate smoother transitions (Roberts et al., 2015, Vohryzek et al., 2020, Deco and Kringelbach, 2017, Kang et al., 2019). Its developing dominance on the metastability temporal dynamic supports the emergence of self-referential processing and higher-order cognition (Knyazev, 2013). Furthermore, we can interpret the trend from more occipital to parietal dominated metastable states as a shift from visual to sensorimotor processes, aligning with the established posterior to frontal developmental axis (Gerván et al., 2017). Metastable state temporal dynamic also initially includes more metastable states defined by local connectivity, suggesting a developmental prioritization of local processing within these regions (such as the thalamus), before switching to longer range and inter-hemispheric processing (Eyre et al., 2021). Preterms at term-equivalent have altered metastable state coverage when compared to full-terms. The slower emergence of whole-brain synchronized states and persisting dominance of occipital-centred states can subsequently be interpreted as possible immature cognitive development.

In preterms followed longitudinally, EEG microstate transition rate increases and duration decreases (Hermans et al., 2024). FMRI dFC state duration continues to decrease postnatally in full-term neonates over the first week after birth (França et al., 2024). Preterms at term-equivalent still have a slower transition rate than full-terms (Adibpour et al., 2025).

In summary, the transition rate between metastable states increases during the equivalent of the third gestational trimester and postnatally in full-terms. Increased transition rate in adults correlates with higher executive function (Medaglia et al., 2018) and working memory (Braun et al., 2021), while a lower transition rate is associated with the autistic-like trait of greater attention to detail (Sase and Kitajo, 2021), and also with brain injuries such as hypoxic ischemic encephalopathy and traumatic brain injury (Hellyer et al., 2015, Khazaei et al., 2023). The increasing transition rate during development could imply a tendency for the brain to "dwell" less in specific metastable states, leading to faster and more flexible responses to external stimuli and cognitive tasks. Preterms at term-equivalent age exhibit slower and less flexible metastable dynamics when compared to full-terms, meaning a potentially slower response to external stimuli and cognitive tasks.

### 3.3 Neonatal signal complexity

In terms of signal complexity and predictability, SE of fMRI BOLD signal decreases over gestation between 37 and 42 weeks (Zhao et al., 2024). In a group of preterms followed longitudinally, MSE of EEG signal increases postnatally until term-equivalent age (Wel et al., 2017), and SE of fMRI BOLD signal continues to increase postnatally in full-terms over the first 5 days after birth (Zhao et al., 2024). Because entropy decreases during gestation but increases after birth, preterms—who have spent more time outside the uterus by term-equivalent age—show higher levels of SE of fMRI BOLD signal compared to full-terms (Zhao et al., 2024). These findings suggest that brain entropy follows distinct developmental trajectories in utero versus ex utero, with preterm birth accelerating the postnatal entropy increase. The Hurst exponent of fMRI BOLD signal is below 0.5 in preterms and above 0.5 in full-terms, reflecting a switch from anti-persistent to persistent and scale-invariant activity (Mella et al., 2024). However, preterms at term-equivalent age still show a lower Hurst exponent, hovering closer to 0.5, compared to full-terms (Mella et al., 2024), suggesting a lag in the maturation of long-range temporal correlations. In contrast, the Hurst exponent of the EEG microstate sequence is higher in preterms (∼0.75) and decreases with increasing PMA until term equivalent age but remains above 0.5 (Hermans et al., 2024), indicating that microstates at preterm are more persistent and their sequence more predictable, despite an underlying anti-persistent hemodynamic signal. Indeed, the preterm EEG is discontinuous and characterised by long-term temporal dependency (Hurst exponent > 0.5) (Hartley et al., 2012).

In summary, signal unpredictability and complexity increase over the equivalent of the third gestational trimester (increase in entropy) with a switch from anti-persistent to persistent activity (increase in Hurst exponent). Brain activity complexity is disrupted in Alzheimer’s disease and positively correlates with higher cognitive performance (Wang et al., 2017) and intelligence (Saxe et al., 2018). The lower brain complexity in early life could therefore indicate lower cognitive performance and immature cognitive functions. Preterms at term-equivalent have higher brain signal unpredictability and complexity (higher entropy) but exhibit a more erratic white-noise-like pattern (Hurst exponent around 0.5) compared to full-terms. This unstructured complex brain activity could therefore imply a delayed cognitive development.

**Fig. 3:**
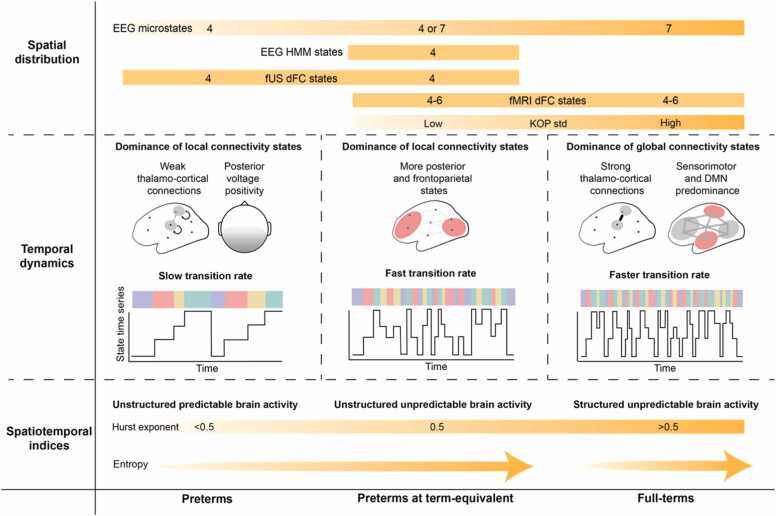
Summary figure of the development of key features of metastability during the equivalent of the third gestational trimester, comparing preterms to preterms at term-equivalent to full-terms**.**

The findings reported in sections IIIA, IIIB and IIIC are summarised in Fig. 3.

### 3.4 Free energy interpretation

**Fig. 4:**
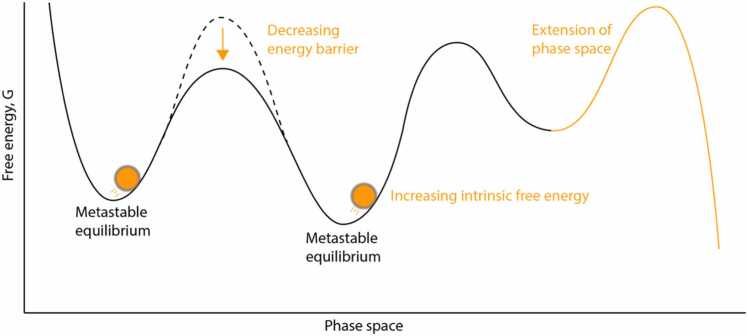
Illustration of a maturing metastable system. Possible interpretations for the changes in metastability observed during development include a lowering of the height of the energy barriers, increase in energy available to the system (in the rolling sphere) and extension of the phase space.

These developmental changes in metastability could be represented as changes in the free energy landscape and/or in free energy available to the brain (Fig. 4). The increasing range of metastable states over the course of the equivalent of the third gestational trimester can be interpreted as an increase in the number and/or change in position of the metastable energy wells in the phase space of the energy landscape. The lower KOP std in preterms at term-equivalent compared to full-terms could represent a limited ability of the brain to scope this phase space.

The increase in transition rate of metastable states could represent a decrease in the energy barriers between energy wells making the transition between certain metastable states easier, leading also to changes in metastable state dominance. That would mean that metastable states representing global thalamo-cortical synchronization are initially surrounded by very high energy barriers, and therefore rarely visited at preterm, and that these barriers then decrease with development allowing the brain to visit these metastable states more frequently at full-term. This change in ease of metastable state transition could be related to the structural arrangement of the brain, which is measured as network controllability or the ability of individual brain regions to drive transitions between functional configurations depending on their structural connections. Densely connected regions can drive more fluid transitions with little energy, while regions with sparse connections require greater energy to overcome resistance to change (Gu et al., 2015), or energy barriers (He et al., 2022). Network controllability of the frontoparietal, somatomotor, and ventral attention networks increases over the equivalent of the third gestational trimester (Sun et al., 2023), potentially helping drive more fluid transitions between metastable states.

Alternatively, the increase in transition rate could also be related to an increase in the brain’s intrinsic free energy required to overcome energy barriers. During the equivalent of the third gestational trimester, the supply of energy is initially constrained by developing vascularisation and is spent on synapse formation and axonal growth following Hebbian principles – i.e. in establishing the position and depth of the metastable states (Tan et al., 2018, Coelho-Santos and Shih, 2020, Cunnane and Crawford, 2014, Harris et al., 2012). With increasing age, there is therefore potentially more energy available to be used for jumping energy barriers and flexible metastability.

Increase in predictability and complexity of brain activity over the equivalent of the third gestational trimester could represent the brain exploring the phase space with increasing predictable and structured trajectories. In preterms at term-equivalent, the brain scopes a limited phase space with a more erratic pattern, potentially because of environmental influences during the period spent outside of the utero.

## 4 Neonatal metastability in different cognitive states

### 4.1 Neonatal metastability across vigilance and behavioural states

Full-terms spend 80 % of their time asleep and preterms up to 90 % (rate which decreases rapidly after birth) (Roffwarg et al., 1966, Trickett et al., 2022) therefore are often in this state during research studies, including the metastability work described above. Preterms and full-terms also have different sleep state organisations to consider (Ryan et al., 2023). However, in adults, vigilance states exhibit distinct metastable patterns, with wakefulness showing faster dynamics than sleep, which gradually slow down as sleep deepens (Wiemers et al., 2023, Brodbeck et al., 2012, Cavanna et al., 2018, Ipiña et al., 2020). Therefore, vigilance is a factor to consider when describing metastability across development.

There is some evidence of changing metastable dynamics in different states of neonatal sleep. While quiet sleep (QS) and active sleep (AS) exhibit the same microstates, they represent less of the signal in AS than QS (Khazaei et al., 2021; Adibpour et al., 2025). AS would perhaps benefit from being characterised by more microstates than QS, which would translate into an extended phase space on the energy landscape. QS is associated with the dominance of HMM states characterised by brain-wide neural synchronization and stronger fronto-temporal activity compared to AS (Khazaei et al., 2023). The cognitive interpretation of this is that QS may prioritize stabilizing and organizing large-scale network structures, supporting the development of integrative functions, while AS might focus more on refining localized circuits. Changes in temporal dynamics between AS and QS, however are inconclusive as different studies report either increase or decrease in metastable state durations and frequency of appearance (Hermans et al., 2024, Khazaei et al., 2021). Therefore, we do not know if the available free energy or height of energy barriers changes between neonatal sleep states. The decrease in Hurst exponent of the microstate sequence with PMA is particularly steep in QS. This might be related to the maturation of EEG during QS from discontinuous burst-suppression pattern, showing very strong temporal dependence and long-term correlations, to a more continuous and variable pattern, decreasing long-term correlations (Dereymaeker et al., 2016, Hermans et al., 2024).

### 4.2 Neonatal metastability in response to external perturbations

Sensory stimuli play a key role in perturbing metastable brain dynamics, often acting as critical disruptors that trigger metastable state transitions (L. Mazzucato et al., 2019; Mazzucato et al., 2015, Mazzucato et al., 2016; La Camera et al., 2019; Jones et al., 2007). Rather than simply evoking isolated responses, sensory inputs are integrated into ongoing spontaneous brain activity, producing complex and context-dependent neural dynamics (Shanahan, 2010). This integration underscores the fact that brain activity cannot be strictly divided into evoked (stimulus-driven) and spontaneous; both must interact to generate an appropriate response (Friston et al., 2012). Sensory stimulation provides the energy perturbation required to overcome the energy barriers that keep the system on a local stable state initiating a selective sequence of metastable state transitions compared to resting state, until all energy dissipates, and dynamics return to the baseline regime (Deco and Hugues, 2012; B.J. He, 2011).

Evidence of neonatal metastability perturbed by sensory input is limited to nociceptive stimuli. In both preterms and full-terms, a single brief tissue-damaging (noxious) heel lance initiate the sequential engagement of a series of microstates which lasts at least 1.5 seconds suggesting that even in preterms, the brain is capable of metastable state transitions in response to the environment (Rupawala et al., 2023, Bucsea et al., 2023). However, the exact same noxious stimulus engages different sequences of EEG microstates in preterms and full-terms (Bucsea et al., 2023). Moreover, the repetition of a noxious stimulus dampens the engagement of initial microstates in full-terms, but not in preterms (Rupawala et al., 2023), where the sequence of metastable states is virtually unchanged following consecutive identical stimuli. This could be related to lower entropy at younger ages representing predictable and less adaptable brain dynamics. The functional significance of these metastable state transitions can be inferred from their relationship with observable behaviours or contextual factors. Comparing the sequence of microstates elicited by the heel lance in a group of full-term (or preterm) neonates who had a strong behavioural response to the stimulus to a group who did not, reveal two interleaved microstate sub-sequences, one which changed together with the magnitude of the behavioural response and one which was independent. This highlighted that the noxious-evoked microstate sequence does not necessarily represent a serial activation of cortical areas but might reflect also parallel processing streams (Bucsea et al., 2023).

## 5 Gaps, challenges and future directions

In general, whether in neonates or adults, metastability is studied across a wide range of imaging modalities and analysis methodologies, making it challenging to achieve consistency and comparability across studies. While there are other tools that are linked to or define metastability, such as brain criticality (Deco and Jirsa, 2012), we restrict this review to those present in the neonatal development literature, which include KOP std, entropy, scale-invariance, as well as spatiotemporal clustering of fMRI, fUS, EEG signal connectivity and/or magnitude. They provided a comprehensive view to metastability, tackling different aspects of brain metastable dynamics. However, differences across modalities, analyses and metrics can complicate efforts to synthesize findings, particularly in a relatively new field such as developmental metastability.

Neonatal imaging introduces additional unique technical and physiological challenges. Neonates are prone to head movements, making the acquisition of clean datasets difficult, particularly in fMRI, which could lead to the extraction of artefact-ridden or non-physiological metastable states (Kim et al., 2023). Existing standardized pipelines and frameworks for analysing metastability are often optimized for adult data and may require adaptation for neonatal datasets. We refer the reader to another review for more in-depth discussion of these modality-level obstacles and possible solutions in imaging the neonatal population (Margolis et al., 2025). Careless consideration of these differences and challenges may lead to inconsistent findings and interpretations of developing metastability.

In this review, we discussed the metastable states studied so far in the context of brain development, including fMRI dFC states, fUS dFC states, EEG HMM states, and EEG microstates. However, neonatal metastability could also be explored using other modalities providing access to other contexts or functional scales. For example, functional Near-Infrared Spectroscopy (fNIRS), a non-invasive, portable modality, could be a viable alternative to fMRI for studying hemodynamic metastability in naturalistic environments, albeit with limited spatial coverage (Zhang and Zhu, 2020). Magnetoencephalography (MEG) directly measures neural activity with better spatial resolution than EEG, which could then complement EEG microstate analyses (Tewarie et al., 2023, Baker et al., 2014). While each modality captures different aspects of metastability (e.g., signal magnitude vs. connectivity, spatial vs temporal precision), future studies could leverage multimodal imaging, such as simultaneous EEG-fMRI or EEG-fNIRS, to connect fine-scale temporal patterns (e.g., microstates) with spatially distributed dFC states (Zhang and Zhu, 2020, Lehmann, 2010). These multimodal approaches could provide a more comprehensive view of neonatal metastability and bridge the magnitude and connectivity aspects.

Consistency in the extraction and characterization of metastable states, within each analysis pipeline, is critical for the field’s growth. For example, there are contrasting evidence about whether the range of metastable states in preterms at term-equivalent is limited compared to full-term controls. Additionally, there are inconclusive results on the increase or decrease in metastable state durations and frequency of appearance between AS and QS. In adults, repeated analyses across multiple studies have established stable templates and meta-criteria for the selection of number of metastable states, particularly for EEG microstates (Koenig et al., 2023). Similar efforts are needed for neonates to establish developmental templates for each imaging modality and measure.

There is still limited evidence regarding the cognitive and functional significance of neonatal metastability and metastable states. This association requires wider study design manipulations addressing specific aspects of neonatal perception and behaviour. For example, while we reviewed changes in metastability following nociceptive stimuli, it would be useful to analyse metastability in other sensory modalities, such as touch and sound. The role of energetic and structural constraints (e.g., immature vascularization and myelination) in shaping neonatal metastability has also been discussed but not directly tested. Studies combining advanced imaging techniques like MRS with fMRI or EEG could provide insights into the metabolic demands of metastable dynamics. Similarly, diffusion-weighted imaging (DWI) could be used to assess how structural connectivity changes support the maturation of metastable states.

Currently, there is also a lack of studies examining fetal metastability. While preterms are often considered a proxy for late-gestation development, this approach has inherent limitations due to environmental factors (e.g., NICU care) influencing preterm brain development. Investigating metastability in the prenatal period using in utero imaging (e.g., fetal fMRI or fUS) presents an opportunity to better understand the emergence of dynamic brain organization during gestation. However, this approach faces significant technical challenges, including fetal and maternal motion, lower spatial and temporal resolution, and the difficulty of extracting reliable dynamic patterns from these scans.

Caution is needed when interpreting claims of 'more' or 'less' metastability, as such descriptions can be vague and potentially misleading. In most cases, these terms implicitly refer to greater or lesser flexibility in brain dynamics rather than a fundamental shift in metastability itself. Metastability is best understood as a point on a spectrum—the brain is either metastable or not—but within this framework, it can exhibit a broader or narrower range of states, as well as faster or slower transitions between them. In the context of early brain development, the neonatal brain should still be considered metastable, but with a different repertoire of states and generally slower dynamics compared to later developmental stages. Future research should aim for precise descriptions of how specific aspects of metastable dynamics change over the equivalent of the third gestational trimester rather than broadly categorizing them as increasing or decreasing.

We aim for this review to provide a comprehensive synthesis of studies on neonatal metastability. However, a key limitation lies in the variability of terminology used across studies, with concepts such as 'transient states' or 'brain dynamics' often describing similar phenomena. This inconsistency makes systematic searches and comparisons challenging. To facilitate future research and reviews, we encourage the adoption of 'metastability' as a standard term when referring to the transient dynamics of functional brain activity.

To address these gaps and challenges, we propose several future directions:

1. Standardization and Validation: Establish standardized pipelines and developmental templates for different imaging modalities and measures of neonatal metastability.
2. Multimodal Imaging: Explore the use of multimodal approaches (e.g., EEG-fMRI, EEG-fNIRS) to capture complementary aspects of neonatal metastability (spatial vs. temporal), as well as to provide insight into metabolic and structural demands of metastable dynamics.
3. Longitudinal Studies: Conduct longitudinal studies to track the developmental trajectory of metastability, separating the effects of maturation from environmental influences (e.g., preterm care) and providing a better understanding of the range of available metastable states.
4. Prenatal Imaging: Further develop and refine fetal imaging techniques to study in utero metastability.
5. Link to Function and Cognition: Investigate how changes in metastability relate to functional and cognitive outcomes in neonates, including sensory and behavioural responses. For instance, studies could investigate whether neonates with more diverse metastable states show better early sensory processing or greater adaptive responses to environmental stimuli.

## 6 Conclusion

In summary, metastability, a concept borrowed from dynamical systems theory, can be applied to functional data from the neonatal brain to model their development. In this review, we first define the most common metastability metrics that cover the core aspects of metastability: spatial distribution and temporal dynamics of metastable states, which include functional integration and segregation, signal predictability and complexity. We then review the evidence of developmental changes in these core features during the equivalent of the third gestational trimester, comparing preterms to full-terms, following preterms longitudinally and comparing preterms at term-equivalent to full-terms. Reports use different methods, metrics, and age groupings to describe metastability changes in early life, making it difficult to identify clear developmental patterns in functional dynamics. Nevertheless, we conclude that evidence suggests that neonatal metastability development - described in terms of spatial distributions of signal magnitude or connectivity - reveals increasing ability for integration-segregation and expansion of the range of metastable states, faster metastable state transitions, enhanced signal complexity and patterned unpredictability. These maturational changes are likely underpinned by structural and functional changes allowing for low energy transitions and increase flexibility. The shifting excitation-inhibition balance likely allows for more controlled transitions avoiding the system to collapse into temporally persistent states dominated by recurrent excitatory networks. The increase in structural and functional connectivity could be related to a decrease in the energy required to move between states, while the increase in axonal growth, dendritic arborization and synaptogenesis could be related to the increase in range of metastable states and synchronization levels. We propose to model this development in metastability as changes in the free energy landscape or energy available to the brain. Finally, we suggest that this evolving metastability may reflect the maturation of flexible cognitive functions and sensory adaptation, while also being vulnerable to preterm birth.

## Funding

JC is supported by the 10.13039/501100000268Biotechnology and Biological Sciences Research Council [grant number BB/T008709/1]. LF is supported by the 10.13039/501100000265Medical Research Council [grant number MR/X010716/1], and the 10.13039/501100009187Medical Research Foundation [grant number MRF-160–0012-ELP-FABR-C0841].

## Declaration of Competing Interest

The authors declare that they have no known competing financial interests or personal relationships that could have appeared to influence the work reported in this paper.
